# Characterization of Carbonation Curing Influence on Nonlinear Ultrasonic Response and Mechanical Performance of Mortar

**DOI:** 10.3390/ma19050874

**Published:** 2026-02-26

**Authors:** Shruti Singh, Hang Zeng, Umar Amjad, Hee-Jeong Kim, Tribikram Kundu

**Affiliations:** 1 Department of Civil and Architectural Engineering and Mechanics, The University of Arizona, Tucson, AZ 85721, USA; shrutis@arizona.edu (S.S.); hangzeng@arizona.edu (H.Z.); 2 Center for Advanced Materials, Qatar University, Doha P.O. Box 2713, Qatar; umaramjad@qu.edu.qa; 3 Department of Materials Science and Engineering, University of Arizona, Tucson, AZ 85721, USA; 4 Department of Aerospace and Mechanical Engineering, University of Arizona, Tucson, AZ 85721, USA

**Keywords:** nonlinear ultrasonics, carbonation curing, cement mortar, CO_2_ sequestration, ultrasonic sensing, compressive strength, microstructural monitoring

## Abstract

The cement industry is a major contributor to global CO_2_ emissions, creating a need for monitoring techniques that support carbon capture strategies while assessing material performance. This study investigates the accelerated carbonation curing of cement mortar using linear and nonlinear ultrasonic sensing methods, alongside mechanical and gravimetric measurements. Mortar specimens were carbonated for 1–28 days and evaluated using ultrasonic pulse velocity (UPV), the Sideband Peak Count Index (SPC-I) for nonlinear ultrasonic response, compressive strength testing, and mass-based CO_2_ uptake analysis. UPV showed sensitivity primarily to bulk material changes, with comparatively less distinction among the observed responses during carbonation curing. In contrast, the SPC-I captured distinct nonlinear responses associated with matrix evolution. Early-age carbonation (<7 days) produced increased nonlinearity, attributed to shrinkage-induced microcracking, whereas extended curing led to reduced SPC-I values, consistent with carbonation curing age. These trends exhibited an inverse correlation with compressive strength, which increased by up to 38.9% on the 28th day compared to the control specimens. Gravimetric analysis confirmed effective CO_2_ sequestration, with average specimen mass gains reaching 2.62%. The findings demonstrate that nonlinear ultrasonic sensing provides a sensitive, nondestructive approach for monitoring carbonation curing and linking acoustic signatures to mechanical performance and carbon uptake in cement-based materials.

## 1. Introduction

The cement and concrete industries are among the largest industrial sources of anthropogenic carbon emissions, contributing an estimated 7–8% of global CO_2_ output, primarily through limestone calcination during clinker production [1,2]. Mitigating this footprint requires scalable strategies that simultaneously advance performance and sustainability. Carbonation curing has emerged as a promising approach that not only enables direct sequestration of CO_2_ but also enhances the microstructural and mechanical properties of cementitious composites [3,4,5].

Carbonation curing involves the accelerated reaction of CO_2_ with hydration products, most notably portlandite (Ca(OH)_2_) and calcium silicate hydrate (C–S–H), yielding calcium carbonate (CaCO_3_) and silica-rich gels [6,7].

These reactions can be generalized as follows:

Formation of calcium carbonate from portlandite [8]:

$$\mathrm{Ca}{\left(\mathrm{OH}\right)}_{2}\;\left(s\right)+{\mathrm{CO}}_{2}\;\left(g\right)\;\to \;{\mathrm{CaCO}}_{3}\;\left(s\right)+H_{2}O\;\left(l\right)$$

The product of this reaction, calcium carbonate (CaCO_3_), typically precipitates as calcite, a crystalline mineral with greater density and intrinsic stiffness than the reactant portlandite. Critically, this transformation is accompanied by an increase in solid volume; the consumption of one mole of Ca(OH)_2_ (molar volume ~33.1 cm^3^/mol) produces one mole of CaCO_3_ (molar volume ~36.9 cm^3^/mol).

Furthermore, the principal binding phase, calcium silicate hydrate (C-S-H), can also undergo carbonation. This process involves the decalcification of the C-S-H gel, resulting in the formation of additional CaCO_3_ and a silica-rich hydrogel (SiO_2_⋅nH2O):

Carbonation of calcium silicate hydrates [8]:

$$C_{x}-S_{y}-H_{z}+{\mathrm{CO}}_{2}\;\to \;{\mathrm{CaCO}}_{3}+{\mathrm{SiO}}_{2}\cdot {\mathrm{nH}}_{2}O$$

The precipitation of CaCO_3_ within pore spaces results in microstructural densification, pore refinement, and volume expansion, which reduce porosity and improve compressive strength, particularly at early ages [4,6,9]. However, the carbonation process is not unidirectional: shrinkage-induced microcracking, local heterogeneity, and evolving pore anisotropy can generate competing effects that complicate both performance outcomes and nondestructive monitoring [10,11].

Conventional nondestructive evaluation (NDE) of carbonation curing has relied heavily on ultrasonic pulse velocity (UPV), standardized in ASTM C597 [12], where elastic wave velocity is interpreted as an indirect indicator of density and quality [13,14]. While UPV has been widely applied, evidence suggests that it is often inadequate for carbonation monitoring. Instead of showing the expected monotonic increase with matrix densification, UPV trends frequently fluctuate or decline during prolonged carbonation, reflecting the competing influences of crack initiation, pore morphology, and scattering effects [6,9,15]. These limitations underscore the insufficiency of linear ultrasonic methods in detecting subtle microstructural transformations during carbonation curing.

To address these shortcomings, nonlinear ultrasonics has emerged as a more sensitive diagnostic tool. Unlike linear methods that measure bulk elastic wave velocity, nonlinear approaches exploit interactions between propagating waves and microstructural features such as pores, interfaces, and microcracks that act as nonlinear scatterers [15,16,17]. Among various nonlinear metrics, the Sideband Peak Count Index (SPC-I) technique has gained attention due to its simplicity, robustness, and strong sensitivity to microstructural nonlinearity [18].

The SPC-I method evaluates the frequency spectrum of ultrasonic signals by quantifying the number of sideband peaks and their strengths arising from frequency mixing effects in nonlinear media. In a perfectly linear material, FFT spectra consist only of input frequencies (with expected attenuation) [19]. By contrast, nonlinear materials generate additional sidebands at intermediate frequencies, with amplitudes typically 1–2% or less of the main spectral peaks. By varying a threshold between 0 and 2% of the dominant amplitude and counting peaks above this level, a peak count–threshold curve is obtained [18]. The SPC Index (SPC-I) is then calculated as the average count over these thresholds, providing a scalar, threshold-independent measure of nonlinearity [19]. Materials with higher microstructural nonlinearity, such as carbonation-induced mortars with microcracks and heterogeneity, are expected to exhibit elevated SPC-I values compared to stable linear media [20].

Concrete’s inherently multiscale heterogeneity, including aggregates, voids, interfacial transition zones (ITZs), and evolving cracks, naturally lends itself to nonlinear elastic characterization [17,21]. Unlike UPV, which assumes homogeneity and linear propagation, nonlinear SPC-based techniques can capture both classical nonlinearity (strain-dependent velocity shifts) and nonclassical hysteresis associated with crack closure, frictional sliding, and pore-interface interactions [20,22]. This makes SPC-I particularly well suited for capturing the evolving stages of carbonation response: an early regime characterized by an elevated SPC-I which is associated with increased material nonlinearity, followed by a later stage where the SPC-I declines in conjunction with matrix evolution during carbonation.

## 2. Research Gap

Despite the well-established benefits of carbonation curing and growing attention to CO_2_ sequestration in cementitious systems [3,4], diagnostic gaps persist in nondestructive monitoring methods. Existing reliance on UPV can lead to misinterpretations of carbonation progress due to its inability to account for competing nonlinear phenomena. Multiparametric approaches using coda wave analysis [23] or acoustic imaging have been explored [24,25], but nonlinear ultrasonic indices remain underutilized in systematic carbonation studies. In particular, the SPC-I has not yet been rigorously evaluated for its sensitivity to carbonation curing, nor correlated with mechanical and gravimetric CO_2_ uptake data.

This study makes the following contributions:

- It demonstrates the limitations of UPV in capturing carbonation-induced transformations, showing non-monotonic trends inconsistent with densification, thus validating the need for nonlinear approaches.
- It establishes the SPC-I as a sensitive nonlinear indicator of carbonation-induced microstructural evolution, capturing competing effects of microcracking, pore refinement, and carbonate precipitation.
- It integrates the SPC-I with gravimetric CO_2_ uptake and compressive strength to provide a multiparametric view of carbonation curing mechanisms.
- It reveals distinct temporal regimes in carbonation curing: an initial nonlinear regime with the elevated SPC-I, followed by a densification regime with reduced nonlinearity, offering a mechanistic phenomenological model.
- It highlights the practical feasibility of the SPC-I as a computationally straightforward, FFT-based diagnostic tool that can be implemented for the real-time, in situ monitoring of carbonation curing in sustainable concrete.

By bridging nonlinear ultrasonic diagnostics with carbonation curing science, this work contributes both methodologically and practically to advancing low-carbon, performance-optimized cementitious materials.

## 3. Materials and Methods

### 3.1. Materials

The mortar specimens were prepared using ordinary Type I/II Portland cement, construction-graded silica sand, and potable tap water. The Portland cement employed in this study conformed to ASTM C150 specifications [26], exhibiting a specific gravity of 3.15 and a Blaine fineness of approximately 350 m^2^/kg. The fine aggregate consisted of a blended fraction of construction-grade sand (sieve sizes #20, #30, and #60), characterized by a specific gravity of 2.60 and satisfying the quality requirements stipulated in ASTM C33 [27]. The aggregates were used in a saturated surface-dry (SSD) condition to minimize variability in water demand.

### 3.2. Mix Proportion and Specimen Preparation

The mixing process was carried out in accordance with ASTM C305-20; Standard practice for mechanical mixing of hydraulic cement pastes and mortars of plastic consistency [28] to ensure the reproducibility and uniformity of the mortar. All raw materials—cement, fine aggregate, and water—were pre-conditioned under identical environmental conditions to maintain consistency and prevent unintended moisture uptake prior to mixing.

Mixing was performed using a standardized mechanical mixer equipped with a flat beater paddle and a stainless-steel bowl. The dry constituents (cement and fine aggregate) were first introduced into the mixing bowl and blended at low speed (140 ± 5 rpm) for 30 s to achieve a homogeneous distribution. Subsequently, the pre-measured mixing water was added gradually over a period of 30 s, while the mixer continued to operate. This was followed by mixing at medium speed (285 ± 10 rpm) for an additional 30 s.

A rest period of 90 s was then observed, during which the material adhering to the bowl walls was scraped down to ensure uniformity. Mixing was completed with a final 60 s cycle at medium speed.

Mortar specimens were prepared with a water-to-cement ratio (w/c) of 0.40, in line with the proportions presented in Table 1. For each test condition, 50 mm × 50 mm × 50 mm cube specimens were cast, with three replicates per curing age to provide statistical reliability. By adhering to ASTM C305 [28], the procedure ensured uniform dispersion of the cementitious matrix and fine aggregates, thereby minimizing variability in the prepared specimens.

Freshly prepared mortar was placed into oiled steel molds (50 mm × 50 mm × 50 mm) in two layers and compacted using a vibrating table to eliminate entrapped air. Specimens were demolded after 8 h of casting. The reference batch was subjected to ambient air curing, while the experimental batches were conditioned by fan-assisted drying at room temperature (23 ± 2 °C) for 4 h to remove surface moisture. Mass measurements were recorded before and after drying to quantify moisture loss. Subsequently, the specimens were transferred to a controlled carbonation chamber, where they were subjected to accelerated carbonation curing under regulated CO_2_ exposure.

### 3.3. Carbonation Protocol

For each designated curing duration, three replicate specimens were subjected to accelerated carbonation treatment. The carbonation regime was implemented for exposure periods of 1, 3, 6, 14, and 28 days (Table 2). Specimens were placed in a sealed carbonation chamber operated under constant pressure, with the chamber maintained at ambient temperature (23 ± 2 °C).

Upon completion of the specified carbonation interval, the specimens were retrieved and subsequently stored under laboratory ambient conditions, alongside the reference (air-cured) batch, until further testing. This ensured consistency in post-curing handling and minimized the influence of uncontrolled environmental variability.

### 3.4. Linear Ultrasonic Testing

The linear ultrasonic response of the mortar specimens was characterized using the Ultrasonic Pulse Velocity (UPV) method in accordance with ASTM C597—Standard Test Method for Pulse Velocity Through Concrete [12]. This nondestructive technique was employed to assess the effect of carbonation curing on the homogeneity and stiffness development of the mortar matrix.

All ultrasonic measurements were conducted on 50 mm cubic specimens, identical to those used for compressive strength testing, in order to maintain geometric consistency across experimental methods. ASTM C597 [12] specifies a minimum transmission path length of approximately 50 mm to ensure reliable time-of-flight determination; the cube configuration adopted in this study, therefore, represents the lower bound of the recommended measurement length. Although UPV values obtained from specimens of this size may not be directly comparable to standard velocity-based quality classifications, the uniform specimen geometry and strictly controlled testing protocol applied to all mixtures ensure consistent measurement conditions. Consequently, any systematic effects associated with specimen size or path length are expected to be constant across all test groups. The UPV results are, therefore, considered robust for comparative evaluation of relative stiffness evolution and carbonation-induced microstructural changes, rather than for absolute material classification.

A commercial UPV device equipped with 54 kHz transducers was used in a direct transmission configuration, with the transmitting and receiving transducers placed on opposite faces of the specimen to ensure maximum signal fidelity. A thin layer of petroleum-based coupling gel was applied at the transducer–specimen interface to minimize interfacial impedance and signal scattering.

Prior to each test, the instrument was calibrated using a standard reference bar provided by the manufacturer. All measurements were conducted under controlled laboratory conditions, with temperature and relative humidity maintained constant throughout the testing campaign to minimize environmental influences on wave propagation.

UPV measurements were performed on the 7th and 28th days of testing for all batches, including both carbonation-cured specimens (1, 3, 6, 14, and 28 days of exposure) and the reference (air-cured) specimens. For each test age, three replicate measurements were taken per specimen, and the average value was reported to ensure reproducibility. The pulse velocity (V) was computed as

$$V=\frac{L}{t}$$

where L is the specimen length (in meters), and t is the measured travel time of the ultrasonic pulse (in seconds). The UPV results were subsequently correlated with compressive strength and carbonation depth to evaluate the linkage between carbonation-induced matrix densification and linear elastic wave propagation [12].

The average velocity from *n* measurements on a specimen was calculated as

$$\bar{V}=\frac{1}{n}\overset{n}{\underset{i=1}{\sum }}V_{i}$$

### 3.5. Nonlinear Ultrasonic Testing

Nonlinear Ultrasonic Testing was conducted on specimens at curing ages of 7, 14 and 28 days, for both the reference samples and those subjected to carbonation curing at different durations (1, 3, 6, 14, and 28 days).

The nonlinear ultrasonic response of the mortar specimens was evaluated using lead zirconate titanate (PZT) transducers mounted in a transmission configuration. Two PZTs were positioned on opposite smooth faces of the 50 mm cube specimens, with one transducer serving as the actuator and the other as the receiver. To ensure efficient wave transmission and minimize interfacial impedance, a thin layer of petroleum jelly was applied at the transducer–specimen interface to eliminate potential air gaps.

The excitation signal consisted of a sinusoidal input pulse with a fundamental frequency of 42 kHz, selected to induce measurable scattering effects within the cementitious matrix and capture the evolution of nonlinear material behavior. To suppress spectral leakage and enhance the accuracy of frequency-domain analysis, the acquired signals were processed using a Hann windowing function, which reduces boundary discontinuities and improves the identification of harmonic and sideband components. Hann windowing enhances the ability to separate weak higher harmonic content from the dominant fundamental frequency, thereby improving sensitivity to subtle nonlinearities in the material response [29,30]. The data were sampled at a frequency of 25 MHz, with a 1000 µs pre-trigger and post-trigger silent period to ensure complete transient capture.

The experimental setup comprised a computer-controlled data acquisition system, integrating an arbitrary waveform generator, a high-speed oscilloscope, and a data logger [19]. The actuator PZT was connected to the output channel of the waveform generator, while the receiver PZT was connected to the oscilloscope input. Transient signals were normalized to their peak amplitude prior to analysis to facilitate comparative evaluation across specimens [19].

**Figure 1:**
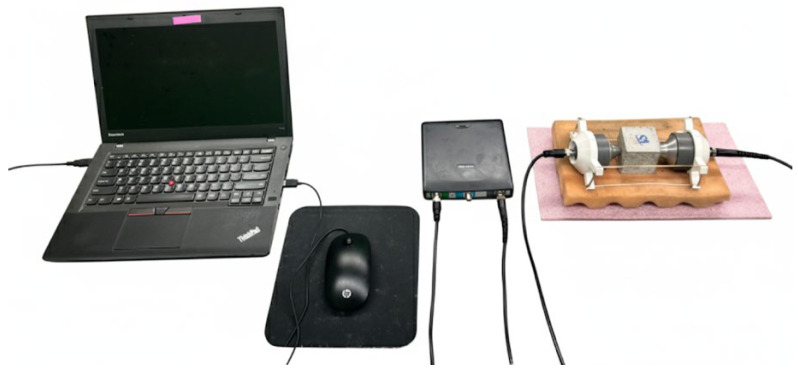
Computer with data acquisition system, oscilloscope, and the specimen with ultrasonic transducers used in present study to measure nonlinear ultrasonic parameters for concrete cube specimens.

Post-processing of the signals was performed in both the frequency and time–frequency domains to extract nonlinear features. The nonlinear response was quantified using the Sideband Peak Count Index (SPC-I), a metric sensitive to the emergence of sidebands generated by microcrack closure, pore refinement, and matrix densification during carbonation curing. Different components of the experimental setup are shown in Figure 1.

### 3.6. Gravimetric Analysis Due to Carbonation

The specimens were weighed both before and after carbonation curing to quantify changes in mass associated with microstructural densification. During carbonation, calcium hydroxide and other hydration products react with carbon dioxide to form stable calcium carbonate phases (e.g., calcite, aragonite, and vaterite), which progressively fill pore spaces and reduce porosity [31]. This process leads to an overall increase in specimen mass at early ages due to the solid reaction products occupying a larger molar volume compared to the consumed reactants. The percentage weight change was determined for each curing duration as the average of three replicate specimens. Such mass gain has been widely reported in the literature as a characteristic indicator of early-age carbonation curing [3,32,33].

While advanced microstructural characterization techniques such as XRD, SEM, and optical microscopy can provide direct evidence of carbonation-induced phase and morphological changes, the present study was designed to prioritize mechanical performance and ultrasonic response as functional indicators of material behavior. The established literature has extensively validated carbonation-induced microstructural changes using such methods, including phenolphthalein-based depth visualization and spectroscopic techniques [34,35], as well as image-processing approaches for carbonation assessment [36].

Therefore, rather than reiterating well-established characterization approaches, this study employed gravimetric CO_2_ uptake as a quantitative proxy for carbonation progression, consistent with previously reported correlations between CO_2_ consumption and microstructural transformation [36].

### 3.7. Compressive Strength Test

The compressive strength of mortar specimens was evaluated in accordance with ASTM C109/C109M—Standard Test Method for Compressive Strength of Hydraulic Cement Mortars [37]. For each mix proportion, three cube specimens measuring 50 mm × 50 mm × 50 mm were tested at both 7 and 28 days of curing to ensure accuracy and statistical reliability of the results.

Prior to testing, the specimens were removed from the curing environment, the surfaces were wiped clean of excess moisture, and the dimensions were verified to confirm compliance with the standard. The specimens were then positioned centrally between the platens of a calibrated compression testing machine. Load was applied continuously and without shock at a controlled rate of 0.9 ± 0.2 kN/s, as prescribed by ASTM C109 [37]. The maximum load carried by each specimen at failure was recorded, and the compressive strength was calculated by dividing this load by the cross-sectional area of the specimen.

The average compressive strength of the three specimens was reported as the representative value for each batch and curing age, while the individual specimen results were also noted to assess variability. The selection of 7 and 28 days as testing ages was made to capture both early-age strength development, which is sensitive to microstructural modifications induced by carbonation curing, and the standard benchmark strength at 28 days, which is widely recognized as a reference age for evaluating the long-term mechanical performance of cementitious systems [38].

## 4. Results

### 4.1. Linear Ultrasonic Response

**Figure 2:**
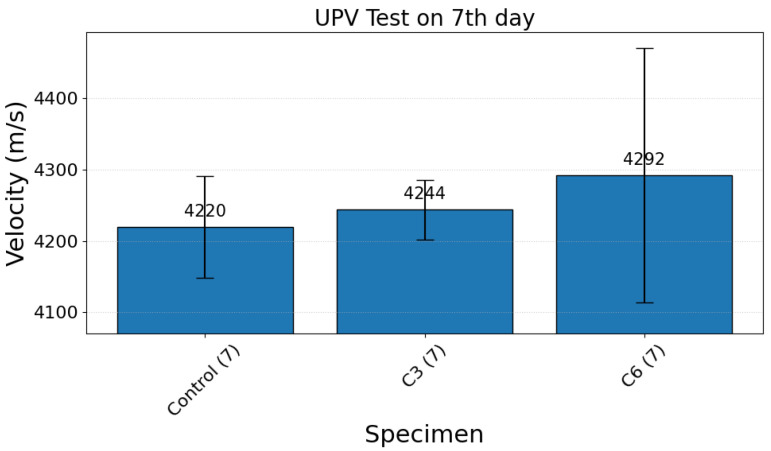
Ultrasonic pulse velocity on the 7th day for the reference (or control specimen) and the carbonation-cured specimens.

**Figure 3:**
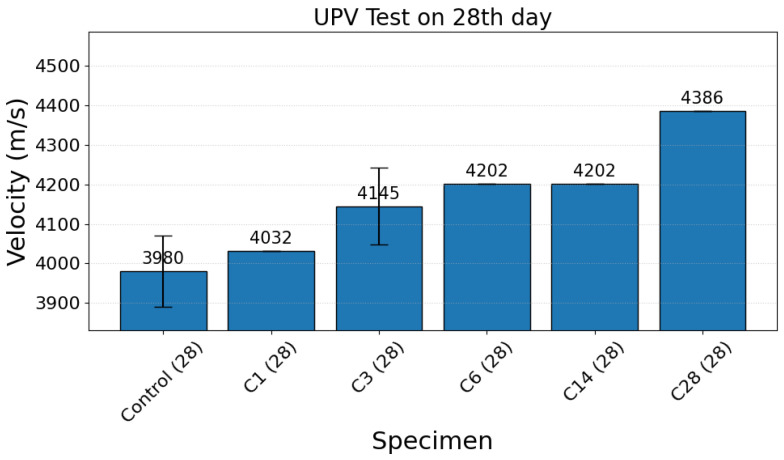
Ultrasonic pulse velocity on the 28th day for the reference (or control specimen) and the 1st, 3rd, 6th, 14th and 28th days for the carbonation-cured specimens.

Ultrasonic pulse velocity (UPV) measurements were used to assess changes in bulk elastic wave speed resulting from accelerated carbonation curing. Mean UPV values (±standard deviation) for the tested batches at 7 and 28 days are presented in Figure 2 and Figure 3, respectively. The UPV data show an overall increase with carbonation exposure time at both ages, although the progression is nonlinear and includes an intermediate saturation regime.

At 7 days, the control (air-cured) specimens exhibited an average UPV of 4219.86 m/s. Carbonation-treated specimens showed slightly higher velocities: 4243.70 m/s for C3 and 4292.35 m/s for C6 (Figure 2). These modest increases at an early age are consistent with initial matrix stiffening caused by early carbonate precipitation and partial pore refinement. The relatively large scatter observed for the C6 group indicates greater sample-to-sample variability, consistent with competing early-age mechanisms (local densification vs. shrinkage-related microcracking) that can increase heterogeneity even while average stiffness rises.

On the 28th day, UPV increased progressively with carbonation duration across the full set of exposures. The mean velocities for the control specimen are 3980.16 m/s, C1 4032.26 m/s, C3 4145.21 m/s, C6 4201.68 m/s, C14 4201.68 m/s, and C28 4385.96 m/s (Figure 3). The data show a clear upward trend from the control to the longest carbonation exposure; however, the response is not strictly proportional to exposure time. In particular, the C6 and C14 specimens have essentially the same mean UPV values (plateau), while the C28 specimens record the largest increase, indicating substantial bulk stiffening under prolonged carbonation.

The observed increases in UPV with carbonation exposure are broadly consistent with the expected effect of carbonate precipitation and pore filling on effective elastic moduli [39]. Nonetheless, the nonlinear progression (growth phases and intermediate saturation) highlights that UPV, being primarily sensitive to bulk longitudinal stiffness, does not uniquely resolve the competing microstructural processes that accompany carbonation (e.g., localized crack formation, anisotropic pore refinement, and heterogeneous carbonation fronts). Standardized UPV measurements (ASTM C597 [12]; EN 12504-4 [40]) remain useful for assessing global material quality, but they are inherently limited when used alone to monitor subtle or spatially non-uniform microstructural transformations induced by carbonation.

Recent studies indicate that complementary wave-based diagnostics (for example, coda-wave analysis) can provide a more direct measure of scattering and multiple-scattering effects that accompany pore structure changes and microcracking and, therefore, may correlate more linearly with carbonation depth than direct UPV measurements alone [23]. In the current work, the UPV results should, therefore, be interpreted as evidence of overall bulk stiffening with carbonation exposure but not as a comprehensive proxy for microstructural damage or carbonation depth. This limitation motivates the use of nonlinear ultrasonic indices (SPC-I) and gravimetric and mechanical tests presented elsewhere in this manuscript to obtain a multiparametric view of carbonation-induced evolution.

### 4.2. Nonlinear Ultrasonic Response

The Sideband Peak Count Index (SPC-I), which can be sensitive to nonlinear ultrasonic behavior, was employed to observe the microstructural evolution of carbonation-cured cementitious matrices on the 7th, 14th, and 28th days. Across all carbonation regimes, the SPC-I demonstrated clear temporal trends, with values initially elevated at early ages, followed by a progressive decline with curing duration. This evolution is consistent with the transition from a heterogeneous, crack-dominated microstructure to a denser, carbonate-filled matrix.

Unlike the linear ultrasonic pulse velocity (UPV) method, which is governed primarily by bulk elastic moduli and is inherently less sensitive to sub-wavelength scale features, the SPC-I provides enhanced diagnostic resolution by capturing nonlinear scattering processes. UPV in this study displayed less sensitive results with carbonation curing to resolve the complex interplay of densification, microcracking, and pore refinement. By contrast, the SPC-I exploited the spectral characteristics of sideband peaks generated under broadband excitation, yielding quantifiable measures of matrix nonlinearity. These values directly tracked microcrack closure, carbonate precipitation, and evolving pore structures, demonstrating diagnostic sensitivity well beyond that of UPV.

**Figure 4:**
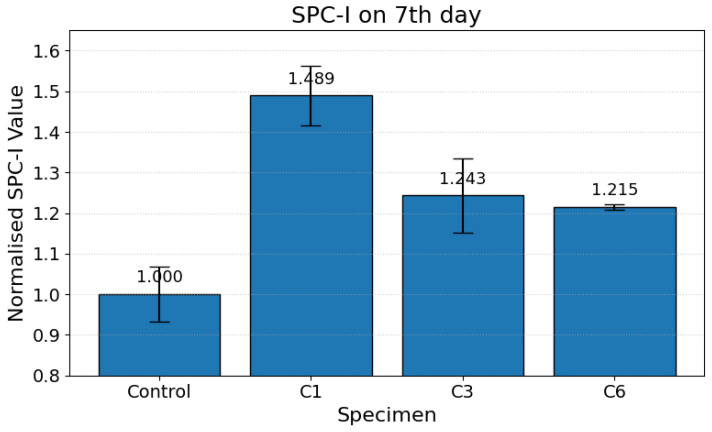
Normalized SPC-I values for carbonation-cured cementitious samples on the 7th day.

On the 7th day, partially carbonated specimens (less than 7 days of carbonation curing) exhibited significantly elevated SPC-I values compared to the control specimens. This pronounced nonlinearity is attributed to early shrinkage-induced microcracking [41] and the formation of a heterogeneous pore network, as shown in Figure 4. Relative to the control specimens, the C1 specimens showed an approximately 49% increase in SPC-I on the 7th day, while C3 and C6 showed approximately 24% and 21% increases, respectively. Micro-voids and -fissures act as nonlinear scatterers, amplifying sideband activity and elevating SPC-I responses.

**Figure 5:**
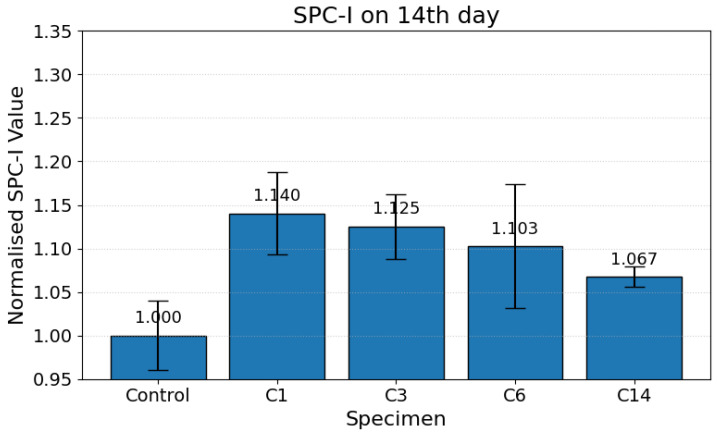
Normalized SPC-I values for carbonation-cured cementitious samples on the 14th day.

By 14 days, the SPC-I values began trending downward across all carbonation levels, as shown in Figure 5. This reduction reflects the progressive precipitation of calcium carbonate phases (e.g., calcite, vaterite, and aragonite) and partial infilling of pores and micro-fissures, which reduce the density of nonlinear scattering sources [42,43]. The reduction in SPC-I values at this stage indicates a transition from a crack-dominated microstructure to one increasingly consolidated by carbonate products.

**Figure 6:**
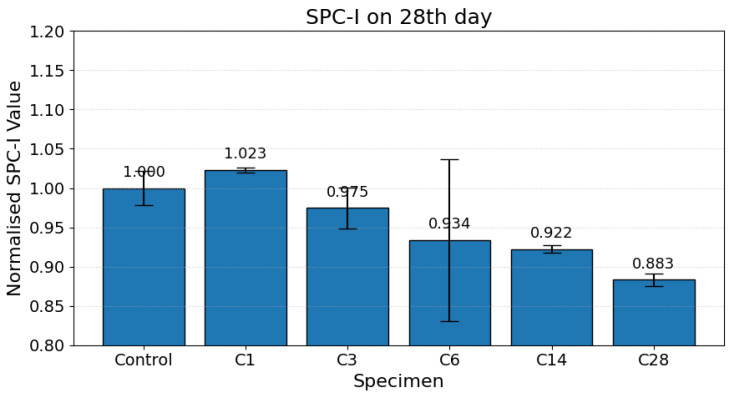
Normalized SPC-I values for carbonation-cured cementitious samples on the 28th day.

On the 28th day, the SPC-I values converged to minimum levels across all curing regimes, evidencing a stiffer, less porous microstructure, as shown in Figure 6. This marked decrease in sideband activity corresponds to maximal densification, pore refinement, and closure of microcrack networks. The C28 specimen shows an approximately 12% reduction in the SPC-I value relative to the control specimen on the 28th day. This mechanistic interpretation is consistent with observations from complementary studies employing mercury intrusion porosimetry, FTIR, and XRD, which confirm carbonate precipitation and pore consolidation under extended carbonation exposure [4,6,9].

Taken together, the SPC-I response delineates two mechanistic regimes of carbonation curing:

Early nonlinear regime (7 days): This is dominated by shrinkage-driven microcrack formation and heterogeneous pore structures, resulting in elevated nonlinearity.

Densification regime (14–28 days): This is characterized by carbonate precipitation, pore filling, and crack closure, leading to suppressed nonlinearity and convergence of SPC-I values across specimens [44].

**Figure 7:**
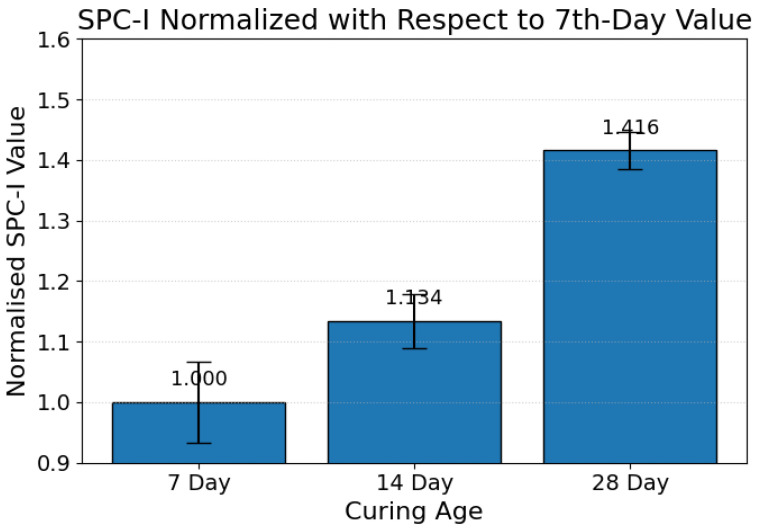
Normalized SPC-I of control specimens on the 7th, 14th, and 28th days.

The evolution of the SPC-I for the uncarbonated (control) specimens across curing ages is shown in Figure 7. Unlike the carbonation-cured specimens, the control samples exhibit a monotonic increase in the normalized SPC-I with time, increasing from 1.00 on the 7th day to 1.134 on the 14th day and reaching 1.416 on the 28th day. Thus, this shows an approximately 13% increase between 7 and 14 days and a further 25% increase from the 14th to 28th day, corresponding to an overall increase of approximately 42% over the 7–28-day interval. The progressive rise in the SPC-I indicates an accumulation of nonlinear scattering sources in the absence of carbonation curing. This behavior is consistent with previously reported nonlinear ultrasonic responses of ambient-cured cementitious materials, where time-dependent drying and shrinkage processes promote the development of microcracks and interfacial discontinuities that enhance material nonlinearity. Interestingly, this trend contrasts sharply with the carbonation-cured specimens, for which the SPC-I decreases with extended curing duration, underscoring the distinct microstructural evolution pathways associated with carbonation-induced densification versus ambient curing. The control response, therefore, provides a critical baseline, confirming that the observed decreasing trend of the SPC-I in carbonated specimens is not an artifact of aging alone but rather a direct consequence of carbonation-driven pore refinement and microcrack closure.

These findings corroborate recent nonlinear ultrasonic studies, which demonstrate that reduced nonlinear indices such as the SPC-I are a reliable phenomenological signature of matrix consolidation, crack closure, and porosity reduction [9,17]. Unlike UPV, which often yields ambiguous or weak correlations due to its reliance on bulk stiffness, the SPC-I offers a threshold-independent, quantitative measure of evolving nonlinearity, directly linking acoustic response to chemical and physical transitions at the mesoscale.

The SPC-I results presented here highlight the diagnostic superiority of nonlinear ultrasonic techniques over conventional UPV for assessing carbonation-induced changes in cementitious materials. By resolving both the shrinkage-induced nonlinear regime at early ages and the densification-driven attenuation at later ages, SPC-I captures the full trajectory of microstructural transformation under carbonation curing. This high-resolution, nondestructive diagnostic technique thus emerges as a critical tool for modern durability research, providing insights into microstructural evolution that cannot be accessed by linear acoustic methods alone [45].

### 4.3. Compressive Strength Test

The effects of early-age accelerated carbonation curing on the mechanical performance of the concrete specimens were quantified by compressive strength testing at 7 and 28 days of curing.

**Figure 8:**
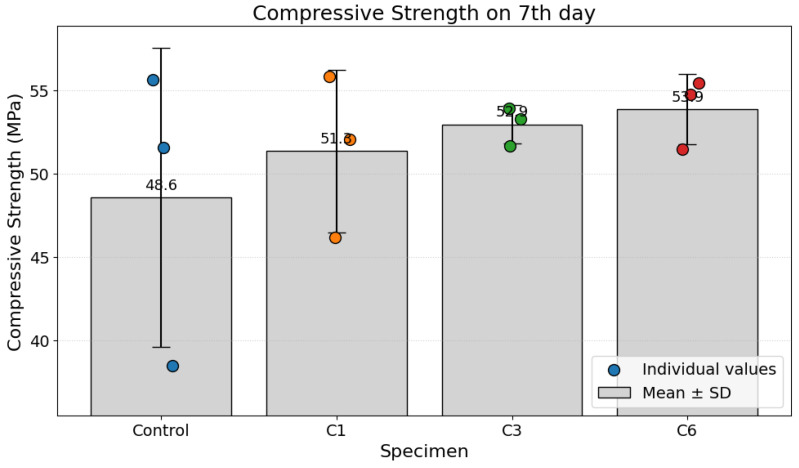
Compressive strength on the 7th day.

The mean compressive strength of the specimens after 7 days is presented in Figure 8. The non-carbonated control specimen exhibited a mean compressive strength of 48.6 MPa. All specimens subjected to carbonation curing demonstrated a marked improvement in strength. A clear positive trend was observed with increased treatment, where the mean strength increased from 51.3 MPa for specimen C1 to 52.9 MPa for C3 and reached a maximum of 53.9 MPa for specimen C6. The C6 specimen thus showed a strength enhancement of 10.9% relative to the control group at this early age. The error bars, representing the standard deviation of the measurements, indicate more consistent performance for the carbonation sample groups compared to the control specimen group.

**Figure 9:**
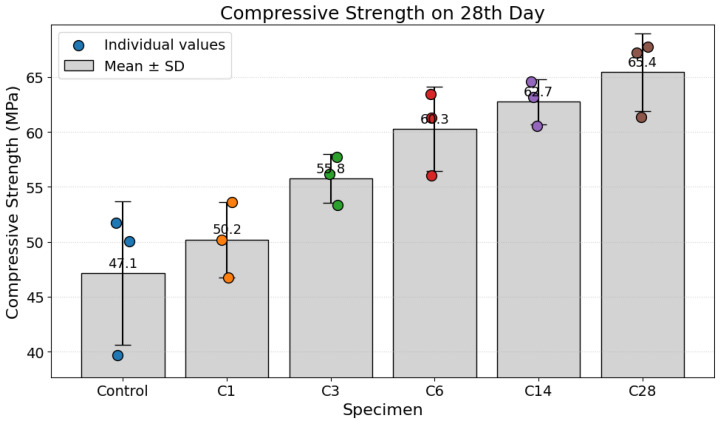
Compressive strength on the 28th day.

The 28th-day compressive strength results, shown in Figure 9, confirm and amplify the trend observed on the 7th day. The control specimen recorded a mean 28th-day strength of 47.1 MPa. In contrast, all carbonated specimens achieved significantly higher strengths. A monotonic relationship between the carbonation treatment and compressive strength is evident, starting at 50.2 MPa for C1 and progressively increasing through C3 (55.8 MPa), C6 (60.3 MPa), and C14 (62.7 MPa). The highest compressive strength of 65.4 MPa was recorded for specimen C28. It represents a substantial strength improvement of 38.9% over the standard-cured control specimen. The results demonstrate that the beneficial effects of carbonation curing are not only maintained but also magnified over longer curing periods.

The experimental results demonstrate a strong positive correlation with R^2^ = 0.91 on the 28th day between early-age carbonation curing and the enhancement of compressive strength. This phenomenon is well documented and is primarily attributed to chemical and microstructural modifications within the cement paste matrix [5].

These findings have significant implications for sustainable engineering practices. The application of carbonation curing presents a compelling strategy for Carbon Capture, Utilization, and Storage (CCUS) within the construction sector. This technology effectively reverses a portion of the emissions-generating process by mineralizing gaseous CO_2_ back into a solid carbonate phase. This process permanently sequesters carbon within the building material, turning a greenhouse gas liability into a performance-enhancing asset. The ability to achieve high early strength, as evidenced by the 7-day results, can also lead to accelerated construction schedules, reduced curing times, and potential cost savings. Therefore, early-age carbonation curing represents a multi-faceted approach to improving both the environmental footprint and the engineering performance of concrete.

A strong inverse correlation is established between the nonlinear ultrasonic response, quantified by the Sideband Peak Count Index (SPC-I), and the material’s compressive strength. At early curing ages (7 days), the elevated SPC-I values are indicative of an “early nonlinear regime” characterized by significant microstructural heterogeneity, including shrinkage-induced microcracking and a poorly consolidated pore network. This state of compromised microstructural integrity, with a high density of inherent flaws that act as stress concentrators, provides a mechanistic explanation for the comparatively modest compressive strength recorded during this initial phase.

As the carbonation process advances to 14 and 28 days, the progressive attenuation of the SPC-I values signifies a transition to a “densification regime.” This trend reflects the precipitation of calcium carbonate phases, leading to systematic pore refinement and the closure of pre-existing micro-fissures. This enhanced matrix consolidation, evidenced by the suppressed nonlinear acoustic response, directly underpins the substantial augmentation of compressive strength observed at later ages. Thus, the SPC-I serves as a sensitive, nondestructive indicator of the microstructural evolution that dictates the material’s macroscopic mechanical behavior, with decreasing nonlinearity being a reliable phenomenological signature of increasing strength and matrix integrity.

### 4.4. Gravimetric Quantification After Carbonation

The degree of carbon dioxide (CO_2_) uptake within the cementitious matrix was quantified via gravimetric analysis [46]. The percentage weight change of the specimens, measured after fan-drying and subsequent exposure to the accelerated carbonation environment, serves as a direct proxy for the extent of reaction and mass of sequestered CO_2_. The results from two separate experimental series are presented below.

**Figure 10:**
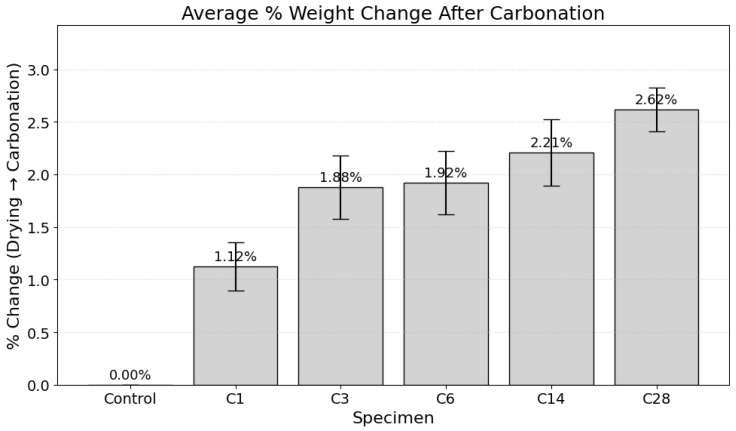
Mean percentage weight gain of specimens subjected to extended carbonation curing regimes.

**Figure 11:**
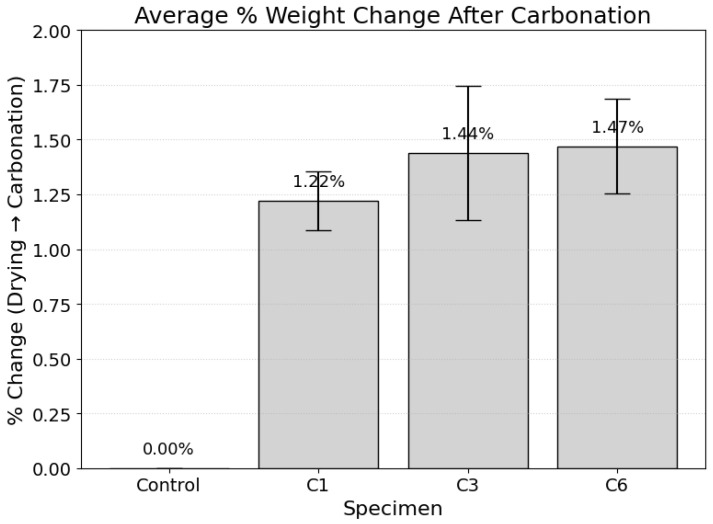
Mean percentage weight gain of specimens subjected to short-term carbonation curing regimes.

In the first series (Figure 10), a clear, dose-dependent relationship between the carbonation curing regime and mass gain was observed. The control specimen, which was not exposed to an enriched CO_2_ environment, exhibited a negligible weight change of 0.00%. In contrast, all carbonated specimens showed a monotonic increase in mass, beginning at 1.12% for the C1 specimen and culminating in a maximum mean weight gain of 2.62% for the C28 specimen. The progressive increase across specimens C3 (1.88%), C6 (1.92%), and C14 (2.21%) further substantiates this trend. A similar pattern was observed in the second experimental series (Figure 11), which corroborates these findings, showing a weight gain from 1.22% for C1 to 1.47% for C6.

This observed mass increase is a direct consequence of the chemical sequestration of gaseous CO_2_ into stable solid carbonate phases. The phenomenon is governed primarily by the stoichiometric reaction of CO_2_ with cement hydration products, most notably portlandite (calcium hydroxide, Ca(OH)_2_) and calcium silicate hydrate (C-S-H) gel [6]. During the carbonation of portlandite (Ca(OH)_2_ + CO_2_ → CaCO_3_ + H_2_O), one mole of gaseous CO_2_ (molar mass ≈ 44.01 g/mol) is incorporated into the solid matrix. Although a molecule of water (molar mass ≈ 18.02 g/mol) is released, the net result is a quantifiable mass increase, which is a well-established indicator of the carbonation degree [4]. Therefore, the gravimetric data provides direct, quantitative evidence of successful CO_2_ sequestration, with the magnitude of uptake correlating directly with the duration of the carbonation curing exposure [46].

## 5. Discussion

This study investigated the influence of accelerated carbonation curing on cementitious matrices using a combined experimental framework incorporating gravimetric CO_2_ uptake, linear ultrasonic pulse velocity (UPV), nonlinear ultrasonic diagnostics (SPC-I), and compressive strength testing. The results provide insight into the coupled physicochemical and mechanical transformations induced by carbonation curing and allow these changes to be interpreted in the context of prior studies and the underlying working hypotheses.

The gravimetric results clearly demonstrate that accelerated carbonation curing enables effective CO_2_ mineralization within the cementitious matrix. The monotonic increase in specimen mass with increasing carbonation duration, reaching up to 2.62% on the 28th day, is consistent with previously reported carbonation reactions involving portlandite and calcium silicate hydrates that form stable calcium carbonate phases. This direct relationship between exposure duration and mass gain supports that carbonation curing functions as a viable carbon sequestration pathway while simultaneously modifying the internal microstructure of the material [47,48].

The linear ultrasonic response, assessed through UPV measurements, revealed an overall increase in wave velocity with carbonation exposure at both early and later ages, indicating progressive bulk stiffening associated with carbonate precipitation and pore refinement. The measured UPV values obtained in this study fall within the range commonly reported for mortar systems in the literature. Previous studies have indicated ultrasonic pulse velocities typically ranging between approximately 3500 and 4500 m/s, depending on curing age, porosity, and mix characteristics [44,49,50,51,52]. For instance, Muhiddin et al. (2024) [52] reported comparable UPV ranges for carbon fiber mortar systems while correlating pulse velocity with mechanical strength. The agreement between the present measurements and published data supports the reliability of the UPV results obtained in this study.

However, the UPV evolution was nonlinear and showed only modest differentiation among intermediate carbonation durations. This behavior aligns with previous studies showing that linear ultrasonic techniques are primarily sensitive to global elastic moduli and assume homogeneous wave propagation. Consequently, UPV is inherently limited in its ability to resolve localized microcracking, pore anisotropy, and heterogeneous carbonation fronts, particularly during early-stage carbonation where competing mechanisms coexist [8,49,50,51,52].

In contrast, the nonlinear ultrasonic Sideband Peak Count Index (SPC-I) proved to be highly sensitive to carbonation-induced microstructural evolution. At early ages, carbonation-cured specimens exhibited substantially elevated SPC-I values relative to the control specimen, reflecting pronounced nonlinearity associated with shrinkage-induced microcracking and heterogeneous pore networks. These observations are consistent with nonlinear ultrasonic studies reporting increased harmonic and sideband generation in the presence of distributed microcracks and interfacial discontinuities. With continued carbonation curing, SPC-I values progressively decreased, indicating attenuation of nonlinear scattering as carbonate precipitation filled pores and promoted microcrack closure. By 28 days, most carbonation-cured specimens exhibited SPC-I values at or below the control, signaling effective matrix consolidation and reduced microstructural nonlinearity.

The contrasting behavior of the uncarbonated control specimens further strengthens this interpretation. The progressive increase in SPC-I observed for the control with curing age is consistent with drying- and shrinkage-related damage accumulation commonly reported for ambient-cured cementitious materials [18]. This divergence confirms that the attenuation of SPC-I in carbonation-cured specimens is not an artifact of aging but rather a direct consequence of carbonation-driven densification and microstructural refinement. It is noted, however, that nonlinear ultrasonic response may also be influenced by additional factors such as microcrack state, interfacial contact conditions, internal stress variations, and moisture distribution.

The compressive strength results corroborate the ultrasonic observations and support the proposed mechanistic interpretation. Carbonation curing produced measurable strength enhancement at both early and later ages, with improvements of 10.9% on the 7th day and 38.9% on the 28th day relative to the control specimen. The inverse correspondence between the SPC-I and compressive strength highlights the ability of nonlinear ultrasonics to capture microstructural changes that directly govern macroscopic mechanical performance. While UPV reflects bulk stiffness development, the SPC-I resolves sub-wavelength damage and consolidation processes that are otherwise obscured in linear ultrasonics-based measurements.

Overall, these findings demonstrate that accelerated carbonation curing induces a transition from an early-age nonlinear regime dominated by microcracking to a later densification regime characterized by pore refinement and crack closure. Within this context, nonlinear ultrasonic diagnostics emerge as a powerful complementary tool to conventional methods, offering enhanced sensitivity to mesoscale features that control durability and strength development. Future work may extend this approach by correlating SPC-I with spatial carbonation depth, microstructural imaging, and long-term durability indicators to further refine nondestructive carbonation monitoring strategies.

## 6. Conclusions

This study demonstrates that accelerated carbonation curing is an effective dual-function strategy that enables CO_2_ uptake while enhancing the mechanical performance of cement-based materials. Gravimetric analysis showed progressive mass gain with carbonation duration, indicating CO_2_ uptake within the cementitious matrix.

Linear ultrasonic pulse velocity measurements captured overall bulk stiffening associated with carbonation curing but exhibited limited sensitivity to intermediate material changes. In contrast, nonlinear ultrasonic diagnostics based on the Sideband Peak Count Index (SPC-I) provided a sensitive, nondestructive indicator of material evolution. Elevated SPC-I values at early ages reflected increased material nonlinearity, while progressive attenuation with curing duration indicated changes in internal structure.

The strong inverse relationship between SPC-I and compressive strength confirms that the nonlinear ultrasonic response is closely associated with mechanical performance development. Early-age nonlinearity corresponded to modest strength development, whereas reduced nonlinearity at later ages coincided with substantial strength enhancement, reaching a 38.9% increase on the 28th day relative to the control.

Taken together, the results demonstrate that carbonation curing leads to measurable CO_2_ uptake and improved mechanical performance, while nonlinear ultrasonic techniques provide enhanced diagnostic sensitivity compared to conventional linear methods. The findings of this study are directly applicable to cement-based materials such as mortar and concrete used in controlled curing environments, including precast components and masonry products. While the present investigation was conducted at the mortar scale, the demonstrated relationship between carbonation curing, strength development, and nonlinear ultrasonic response provides a basis for potential extension to concrete systems. Application to reinforced concrete structures would require further study, considering steel–concrete interaction and field curing conditions. The integration of accelerated carbonation curing with advanced nondestructive evaluation using SPC-I offers a promising pathway toward sustainable, high-performance cementitious materials and may inform future applications of Carbon Capture, Utilization, and Storage (CCUS) strategies in the construction sector.

## Figures and Tables

**Table 1:** Mix design of cementitious specimens subjected to carbonation curing (all quantities in kg/m^3^).

w/c	Water	Cement	Sand	Sand #20	Sand #30	Sand #60
0.4	309.7	744.6	1849.6	1294.8	277.4	277.4

**Table 2:** Nomenclature of specimens.

Sample ID	Carbonation Curing Age (In Days)
Control	0
C1	1
C3	3
C6	6
C14	14
C28	28

## Data Availability

The original contributions presented in this study are included in the article. Further inquiries can be directed to the corresponding authors.

## References

[1] Andrew R.M. (2018). Global CO_2_ emissions from cement production. Earth Syst. Sci. Data.

[2] Scrivener K.L., John V.M., Gartner E.M. (2018). Eco-efficient cements: Potential economically viable solutions for a low-CO_2_ cement-based materials industry. Cem. Concr. Res..

[3] Monkman S., Shao Y. (2006). Assessing the carbonation behavior of cementitious materials. J. Mater. Civ. Eng..

[4] Zhan B.J., Xuan D.X., Poon C.S., Shi C.J. (2019). Mechanism for rapid hardening of cement pastes under coupled CO_2_–water curing regime. Cem. Concr. Compos..

[5] Zhang L., Zha X., Ning J., Li W. (2023). Research status on the application technology of early-age carbon dioxide curing. Buildings.

[6] Morandeau A., Thiéry M., Dangla P. (2014). Investigation of the carbonation mechanism of CH and C–S–H in terms of kinetics, microstructure changes and moisture properties. Cem. Concr. Res..

[7] Bertos M.F., Simons S.J.R., Hills C.D., Carey P.J. (2004). A review of accelerated carbonation technology in the treatment of cement-based materials and sequestration of CO_2_. J. Hazard. Mater..

[8] Bustamante M., Letelier V., Wenzel B., Torres C., Loyola E., Ortega J.M. (2024). Effect of accelerated carbonation on fine cement paste aggregates. Dev. Built Environ..

[9] Villarreal A., Cano-Barria P.F.J., León-Martínez F.M., Medina L., Castellanos F. (2025). Ultrasonic monitoring of carbonation in Portland cements: Nonlinear and linear analysis. arXiv.

[10] Dias R.L., Beltrame N.A.M., Gonzalez J.R., Medeiros-Junior R.A. (2023). Effect of duration and pressure of carbonation curing on the chloride profile in concrete. Rev. IBRACON Estrut. Mater..

[11] Xu Y., Liang X., Wan C., Yang H., Feng X. (2023). Carbonation and related behaviors of hardened cement pastes under different hydration degrees. Cem. Concr. Compos..

[12] (2016). Standard Test Method for Pulse Velocity Through Concrete.

[13] Roobankumar R., SenthilPandian M. (2025). Investigating the correlation between ultrasonic pulse velocity and compressive strength in polyurethane foam concrete. Sci. Rep..

[14] (2013). Nondestructive Test Methods for Evaluation of Concrete in Structures.

[15] Kim J.Y., Jacobs L.J., Qu J. (2006). Nonlinear ultrasonic wave techniques for damage detection. J. Nondestruct. Eval..

[16] Zhang J., Zheng D. (2002). Application of windowing techniques in ultrasonic signal processing for concrete evaluation. NDT E Int..

[17] Bouchaala F., Payan C., Garnier V., Balayssac J.-P. (2011). Carbonation assessment in concrete by nonlinear ultrasound. Cem. Concr. Res..

[18] Alnuaimi H.N., Sasmal S., Amjad U., Nikvar-Hassani A., Zhang L., Kundu T. (2021). Monitoring concrete curing by linear and nonlinear ultrasonic methods. ACI Mater. J..

[19] Alnuaimi H., Amjad U., Russo P., Lopresto V., Kundu T. (2021). Monitoring damage in composite plates from crack initiation to macro-crack propagation combining linear and nonlinear ultrasonic techniques. Struct. Health Monit..

[20] Basu S., Thirumalaiselvi A., Sasmal S., Kundu T. (2021). Nonlinear ultrasonics-based technique for monitoring damage progression in reinforced concrete structures. Ultrasonics.

[21] Mašek J., Miarka P. (2025). Mesoscale FEM model of concrete: Statistical assessment of inherent stress concentrations in dependence on phase heterogeneity. Finite Elem. Anal. Des..

[22] Tang Y., Liu G., Schollbach K., Chen Y., Chen W., Brouwers H.J.H. (2022). Re-cementation effects by carbonation and the pozzolanic reaction on LWAs produced by hydrated cement paste powder. J. Clean. Prod..

[23] Sun X., Yi S., Li X., Cui Y., Zhang M., Jiang T., Wang L., Kong Q. (2024). A novel ultrasonic-velocity-based concrete carbonation monitoring method based on stepwise stretching technique. Struct. Health Monit..

[24] Eiras J.N., Kundu T., Popovics J.S., Monzó J., Borrachero M.V., Payá J. (2016). Effect of carbonation on the linear and nonlinear dynamic properties of cement-based materials. Opt. Eng..

[25] Curis S. (2021). Real-Time In Situ Monitoring of Cement Carbonation with Acoustic System. Master’s Thesis.

[26] (2023). Standard Specification for Portland Cement.

[27] (2023). Standard Specification for Concrete Aggregates.

[28] (2020). Standard Practice for Mechanical Mixing of Hydraulic Cement Pastes and Mortars of Plastic Consistency.

[29] He P. (2004). Simulation of ultrasound pulse propagation in lossy media obeying a frequency power law. IEEE Trans. Ultrason. Ferroelectr. Freq. Control.

[30] Krautkrämer J., Krautkrämer H. (2013). Ultrasonic Testing of Materials.

[31] Zeng H., Ellersick L.F., Baah T.T., Xian X., Kim H. (2025). Carbonation curing for recycling and property enhancement of copper slag-based blended mortar. J. CO_2_ Util..

[32] Zuo W., Zhao X., Luo J., He Z., Zhao J. (2024). Investigating the impact of carbonation curing concentrations on the mechanical properties and microstructure of recycled concrete. J. Build. Eng..

[33] Lamaa G., Duarte A.P.C., Silva R.V., de Brito J. (2023). Carbonation of alkali-activated materials: A review. Materials.

[34] Lo Y., Lee H.M. (2002). Curing effects on carbonation of concrete using a phenolphthalein indicator and Fourier-transform infrared spectroscopy. Build. Environ..

[35] Li B., Tian Y., Zhang G., Liu Y., Feng H., Jin N., Jin X., Wu H., Shao Y., Yan D. (2023). Comparison of detection methods for carbonation depth of concrete. Sci. Rep..

[36] Forsdyke J.C., Lees J.M. (2021). Carbonation depth measurement of concretes exposed to different curing and preconditioning conditions using image-processing tools. Proceedings of the 2nd fib Symposium on Concrete and Concrete Structures, Rome, Italy, 18–19 November 2021.

[37] (2021). Standard Test Method for Compressive Strength of Hydraulic Cement Mortars.

[38] Marikunte S.S., Phelps R.J. (2008). Correlation of compressive strength with ultrasonic pulse velocity for high performance concrete. Proceedings of the Concrete Bridge Conference, St. Louis, MO, SUA, 4–7 May 2008.

[39] Ortega J.M., Miró M., Ibáñez-Gosálvez J., Tenza-Abril A.J. (2023). Non-destructive evaluation of the effects of exposure environment in mortars using non-linear ultrasonic measurements. Dev. Built Environ..

[40] (2004). Testing Concrete in Structures—Part 4: Determination of Ultrasonic Pulse Velocity.

[41] Kim G., Kim J.-Y., Kurtis K.E., Jacobs L.J. (2017). Drying shrinkage in concrete assessed by nonlinear ultrasound. Cem. Concr. Res..

[42] Guan X., Liu S., Feng C., Qiu M. (2016). The hardening behavior of γ-C2S binder using accelerated carbonation. Constr. Build. Mater..

[43] Mu Y., Liu Z., Wang F., Huang X. (2018). Carbonation characteristics of γ-dicalcium silicate for low-carbon building material. Constr. Build. Mater..

[44] Jiang J., Zhang D., Gong F., Zhi D. (2022). Prediction of ultrasonic pulse velocity for cement, mortar, and concrete through a multiscale homogenization approach. Materials.

[45] Hu B., Kundu T. (2025). Probabilistic damage analysis of rebar corrosion in recycled aggregate concrete using sideband intensity-based nonlinear ultrasonic technique. Struct. Health Monit..

[46] Han S.H., Jun Y., Shin T.Y., Kim J.H. (2020). CO_2_ Curing Efficiency for Cement Paste and Mortars Produced by a Low Water-to-Cement Ratio. Materials.

[47] El-Hassan H., Shao Y. (2014). Carbon storage through concrete block carbonation curing. J. Clean Energy Technol..

[48] Gilroy B., Black L., Thompson D., Hogan R., Holmes N. Effects of accelerated carbonation curing on CO_2_ sequestration and compressive strength of concrete masonry units. Proceedings of the Civil Engineering Research in Ireland (CERI) 2020: 27th Conference Proceedings.

[49] International Atomic Energy Agency (2002). Guidebook on Non-Destructive Testing of Concrete Structures (IAEA–TCS–17).

[50] Kim W., Jeong K., Lee T. (2024). Statistical reliability analysis of ultrasonic velocity method for predicting residual strength of high-strength concrete under high-temperature conditions. Materials.

[51] Widodo S., Ma’arif F., Mahardika H. (2022). Correlation of ultrasonic pulse velocity with porosity and compressive strength of mortar with limestone for building quality assessment. UKaRsT.

[52] Muhiddin A.B., Tjaronge M.W., Caronge M.A., Khalid N.H.A. (2024). Reliability assessment of carbon fiber mortar: Combined pulse velocity, point load, and compressive strength tests. Results Eng..

